# Hemangioblastoma masquerading as a ring enhancing lesion in the cerebellum: A case report

**DOI:** 10.1097/MD.0000000000028665

**Published:** 2022-01-21

**Authors:** Li Li, Hui-Min Xie, Seidu A. Richard, Zhigang Lan

**Affiliations:** a Department of Neurosurgery, West China Hospital, Sichuan University, Chengdu, Sichuan, PR China; b Department of Radiology, West China Hospital, Sichuan University, Chengdu, Sichuan, PR China; c Department of Medicine, Princefield University, Ghana, West Africa.

**Keywords:** cerebellum, enhancement, hemangioblastomas, immunohistochemical, radiology, surgery

## Abstract

**Rationale:**

Hemangioblastomas (HGBMs) are very rare, and the cerebellum is usually the most common site of occurrence. HGBMs with ring-enhanced walls are often misdiagnosed as metastases, abscesses, glioblastomas, tuberculomas, and demyelinating diseases. Thus, we present a rare case of HGBM masquerading as a ring-enhancing lesion in the cerebellum.

**Patient concerns:**

We present a 33-year-old female who was admitted to our department because of headaches, unstable walking, and visual loss in both eyes. Cranial nerve examination revealed deficits in cranial nerve II.

**Diagnosis:**

Magnetic resonance imaging revealed 2 cystic lesions in the cerebellum, with irregular ring-enhanced cyst walls composed of smaller nodular parts. Immunohistochemical staining of resected specimens established HGBM.

**Interventions:**

The lesions were completely resected using a right retrosigmoid approach.

**Outcomes:**

Two years of follow-up revealed no recurrence of her symptoms or tumor. She is currently well and performs her daily duties.

**Lessons:**

HGBMs with enhanced cysts are often misdiagnosed by radiology because of their ring-enhanced nature. Computed tomography angiography may be the best modality for differentiating cerebellar HGBM from other ring-enhancing lesions. Surgery is the gold standard of treatment for these lesions.

## 1 Introduction

Hemangioblastomas (HGBMs) are very rare and constitutes about 1.5% to 2.5% of all intracranial tumors and about 7% to 8% of all posterior cranial fossa tumors.^[1–3]^ The cerebellum is the most common site of occurrence.^[4–6]^ HGBMs with ring-enhanced walls are often misdiagnosed as metastases, abscesses, glioblastomas, tuberculomas, and demyelinating diseases.

There are several descriptions of this tumor based on magnetic resonance imaging (MRI) findings.^[4,7–9]^ The most common kind composes of a small nodular mass with a large cyst.^[4,8]^ The 2 rarer types are composed of a solid tumor or a lesion with either a ring-enhanced cystic wall or no enhanced cystic component.^[4,8]^ Surgical resection is the most effective and efficient treatment modality for cerebellar HGBMs with enhanced cystic walls.^[4,8]^ HGBMs with ring-enhanced walls are rare, and to date, only a few cases have been described.^[1,4,8,9]^ Thus, we present a rare case of HGBM masquerading as a ring-enhancing lesion in the cerebellum.

## 2 Case report

A 33-year-old female was admitted to our department because of headache, unstable walking, and visual loss in both eyes. She experienced headaches for a year prior to unstable walking, facial numbness, and visual loss in both eyes for 1 week duration. Her visual loss started with occasional transient amaurosis fugax, which aggravated into total loss of vision. The patient's medical history was unremarkable. General physical examinations did not yield much. Neurological examination revealed deficits in cranial nerve II function. However, all the other cranial nerves were intact. Ophthalmic examination further confirmed deficits in cranial nerve II activity. Routine laboratory investigations revealed that all parameters were within the normal ranges. Chest radiography and electrocardiography revealed no abnormalities.

**Figure 1:**
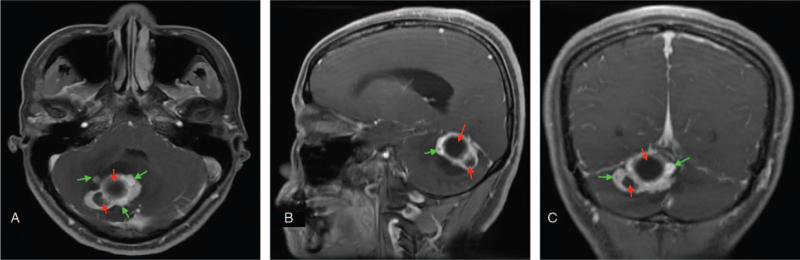
(A–C) Preoperative MRIs showing the two cystic lesions in the cerebellum with irregular ring enhanced cyst walls composed of smaller nodular parts. A = axial, B = sagittal, and C = coronary Red arrow = cystic lesion with ring-enhanced walls. Green arrows indicate tumor nodules. MRI = magnetic resonance imaging.

**Figure 2:**
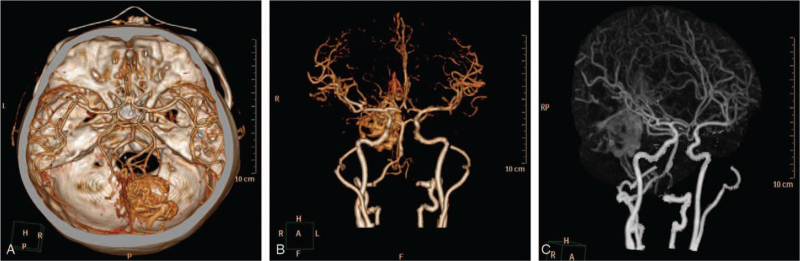
(A–C) Preoperative CTA images showing very rich vascular supply at the tumor bed. CTA = Computed tomography angiography.

MRI revealed two irregular cystic-solid masses in the right cerebellar hemisphere measuring about 3.9 × 3.6 × 3.4 cm and 2.1 × 2.0 × 1.5 cm in diameter **(**Fig. 1A–C**)** The lesions exhibited uneven signal intensities on both the T1 and T2 weighted images. The edges of the lesions were more visible on enhancement imaging and composed of smaller nodular parts. Thus, the lesions showed ring enhancement on contrast-enhanced imaging. The lesions also compressed adjacent structures, thus pushing the cerebellar tonsil to herniate into the spinal canal. In addition, the temporal supraventricular system expanded, but there was no shift in the midline structures. The MRI characteristics of the lesions led us to suspect ring-enhancing lesions such as metastasis, abscess, glioblastoma, tuberculoma, or demyelinating disease. Nevertheless, computed tomography angiography (CTA) revealed that the lesion had a rich vascular supply (Fig. 2A–C) and therefore, may be of vascular origin.

The conflicting radiological imaging findings prompted us to operate on the lesion so that we could establish a definitive diagnosis. The patient was placed on the park bench position with her head fixed in the Mayfield three-key head support system. Routine electromyography and auditory brainstem responses were attached to the patients to monitor the cranial nerves. The lesion was accessed via the right retrosigmoid approach. Intraoperatively, the lesions were cystic, solid, soft, and purplish in color with a very rich blood supply. The borders of the tumors and adjacent brain were unclear. Nevertheless, we performed total resection after meticulous dissection of the adjacent structures and tumor removal. The bone flap was replaced, and the skin was closed after attaining total hemostasis.

**Figure 3:**
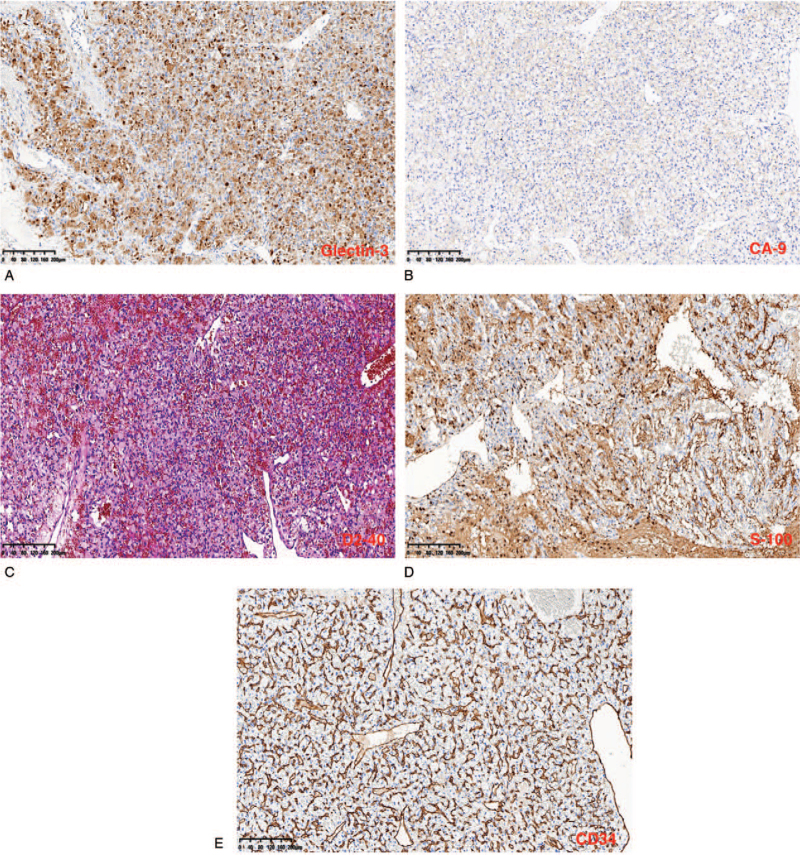
Immunohistochemical stained images showing positivity for glectin-3 = A, CA9 = B, D2-40 = C, S-100 = D, CD34 = E. CA9 = carbonic anhydrase IX.

**Figure 4:**
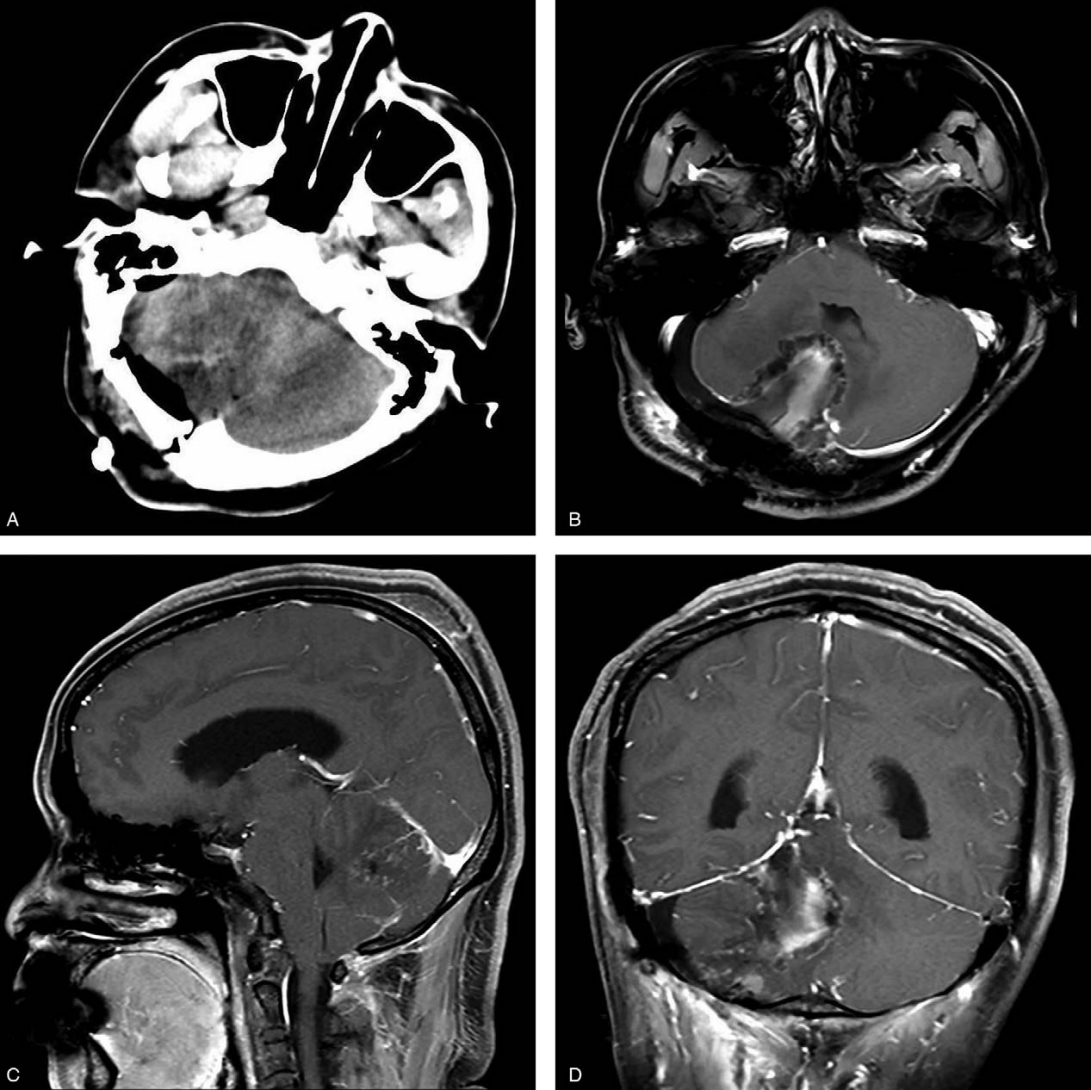
(A–D): Postoperative CT scan and MRIs showing total resection of the tumor. **A**, CT scan; B, axial; C, sagittal; D, coronary. CT = computed tomography, MRI = magnetic resonance imaging.

Immunohistochemical staining of the specimens revealed positivity for glectin-3, carbonic anhydrase IX, D2-40, S-100, CD34, and a 5% Ki 67 index (Fig. 3A–E). Nevertheless, glial fibrillary acidic protein, oligodendrocyte transcription factor, epithelial membrane antigen, inhibin, and phosphoglucomutase-1 tests were negative. These findings were consistent with the diagnosis of HGBM. Postoperative computed tomography (Fig. 4A) and MRI revealed total resection of the tumor (Fig. 4B–D). Her symptoms resolved with no further neurological deficits postoperatively. The patient was discharged home 2 weeks after the operation. Two years of follow-up revealed no recurrence of her symptoms or tumor. She is currently well and performs her daily duties.

## 3 Discussion

HGBMs mostly originate from cerebellar hemispheres in approximately 76% of patients.^[1,10,11]^ Thus, they are the most common primary tumor of the cerebellum in adults.^[1,10]^ These tumors have a male predominance (53.1%) compared with females.^[1]^ They are most frequently detected in patients in their 5th and 6th decades of life, with a mean age of 42.7 years.^[1,10]^ Our patient was a woman, and her age was relatively younger than the mean age. The initial radiological diagnosis was challenging because the lesions were seen with ring-enhanced walls and were therefore mistaken for lesions such as metastasis, abscess, glioblastoma, tuberculoma, or demyelinating disease, which often present as ring-enhancing lesions.

HGBMs are often associated with familial autosomal dominantly inherited Von Hippel–Lindau disease in about 5% to 30% of cases.^[5,11,12]^ In addition, sporadic manifestations of these tumors have been reported, although very rarely.^[1,11,13]^ Our patient did not have Von Hippel–Lindau disease; thus, the etiology was sporadic. The symptomatology of these lesions is often a result of increased intracranial pressure (50.4%), mainly linked to tumor size and/or cyst-related mass effects, if a cystic component is present.^[1,13]^ The most common clinical presentations are headache, vertigo, ataxia, nausea, or vomiting.^[13]^ The main symptoms in our patient were headache, unstable walking, and visual loss in both eyes.

Cerebellar HGBMs often appear as large cysts with small tumor nodules on radiological examination.^[4,8]^ These types are categorized into 2 subtypes: cerebellar HGMBs composed of large cysts with no enhanced cystic walls but with uniformly enhanced tumor nodules, and cerebellar HGBMs composed of large cysts with enhanced cystic walls and tumor nodules.^[4,8]^ The less common types of cerebellar HGBM are often solid tumors.^[4,8]^ These types are further categorized into two subtypes: cerebellar HGMBs composed of multiple solid tumors with homogeneous enhancement, and cerebellar HGBMs composed of solid tumors with a single or multiple cysts.^[4,14]^ The solid portion is often enhanced, whereas the cystic portion is non-enhanced.^[4,14]^

The first type is often associated with surrounding edema, whereas the other 2 types usually present with an obvious mass with no associated edema.^[4,8]^ In addition to the two main tumor types above, the rarest variant of cerebellar HGBMs often presents with an irregular ring-enhanced cyst wall because of cystic nodules.^[4,15]^ On MRI we observed two cystic lesions in the cerebellum, with irregular ring-enhanced cyst walls composed of smaller nodular parts. Thus, our case is one of the rarest presentations of cerebellar HGBMs. CTA was crucial in revealing the rich vascular supply of the lesions and thus provided a clue that the lesions were of vascular origin. In case of a diagnostic dilemma, CTA may be the best modality to differentiate cerebellar HGBM from other ring-enhancing lesions. Notably, solid cerebellar HGBM and nodular cerebellar HGBM with ring-enhanced walls are most often misdiagnosed as lesions above.^[4,15]^

Surgical resection is the most effective and efficient treatment modality for cerebellar HGBMs with an enhanced cystic wall.^[4,14–16]^ For this type of lesion, the tumor must be resected to avoid excessive intraoperative blood loss due to the rich vascular supply of the lesion.^[4,8]^ First, the feeding artery must be occluded to reduce the surface tension of the tumor before occlusion of the draining veins and subsequent removal of the tumor.^[8,17]^ In addition, to avoid tumor recurrence, the wall as well as the solid part of the tumor must be totally resected because the enhanced tumor wall often contains partial tumor cells.^[8,18]^ Although the boards with the lesions and the adjacent brain were unclear, we performed total resection after meticulous dissection of the adjacent structures and removal of the entire tumor. Preoperative embolization with surgical excision is also a safe and successful combination treatment for solid HGBMs of the cerebellum and brainstem.^[12,19]^

Postoperative hemorrhage, hydrocephalus, and pseudomeningocele formation are the most common complications of surgical resection.^[1]^ No postoperative complications were observed. Prognosis and surgical outcomes are generally good after total tumor removal. Nevertheless, Kuharic et al, in a meta-analysis, estimated a total postoperative mortality of 10.3% for cerebellar HGBMs, which was appreciably higher than that reported in earlier studies. Positive immunohistochemical staining for vimentin, vascular endothelial growth factor, neuron-specific enolase, reticulin, CD 56, S-100, and inhibin in decreasing order of frequency is often observed in approximately 80% of patients.^[1]^ We observed positivity for glectin-3, CA9, D2-40, S-100, CD34, and a 5% Ki 67 index which was consistent with the diagnosis of HGBM.

## 4 Conclusion

The initial radiological diagnosis of cerebellar HGBMs is often challenging because these lesions most often exhibit ring-enhancing walls and, therefore, may be mistaken for lesions such as metastasis, abscess, glioblastoma, tuberculoma, or demyelinating disease, which often present as ring-enhancing lesions. CTA may be the best modality for differentiating cerebellar HGBM from other ring-enhancing lesions. Surgery is the gold standard of treatment for these lesions. The tumor must be resected to avoid excessive intraoperative blood loss owing to the rich vascular supply of the lesion.

## Author contributions

**Conceptualization:** Li Li, Hui-Min Xie, Seidu A Richard, Zhigang Lan.

**Data curation:** Li Li, Hui-Min Xie, Seidu A Richard, Zhigang Lan.

**Formal analysis:** Li Li, Hui-Min Xie, Seidu A Richard, Zhigang Lan.

**Funding acquisition:** Zhigang Lan.

**Investigation:** Li Li, Hui-Min Xie, Seidu A Richard, Zhigang Lan.

**Methodology:** Li Li, Hui-Min Xie, Seidu A Richard, Zhigang Lan.

**Resources:** Hui-Min Xie, Zhigang Lan.

**Supervision:** Zhigang Lan.

**Writing – original draft:** Seidu A Richard.

**Writing – review & editing:** Li Li, Hui-Min Xie, Seidu A Richard, Zhigang Lan.
